# Quality of life in people with syndromic heritable thoracic aortic disease and their relatives: a qualitative interview based study

**DOI:** 10.1186/s13023-024-03485-3

**Published:** 2025-01-09

**Authors:** Gry Velvin, Heidi Johansen, Gunnbjørg Aune, Kerstin Fugl-Meyer, Amy Østertun Geirdal

**Affiliations:** 1 Research and Innovation Department, TRS National Resource Centre for Rare Disorders, Sunnaas Rehabilitation Hospital, Nesodden, Norway; 2 Department of Neurobiology, Care Sciences and Society, Division of Family Medicine and Primary Care, Karolinska Institute, Stockholm, Sweden; 3 Department of Social Work, Child Welfare and Social Policy, Faculty of Social Science, Oslo Metropolitan University, Oslo, Norway

**Keywords:** Quality of life, Marfan syndrome, Loeys Dietz syndrome, Vascular Ehlers Danlos syndrome, Syndromic Heritable Thoracic Aorta Disease, Qualitative study

## Abstract

**Introduction:**

The purpose of this study was to investigate perceptions and opinions on what constitutes determinants for quality of life (QoL) in individuals with syndromic Heritable Aortic Disease (sHTAD), utilizing a qualitative study approach. Further to discuss clinical implications and direction for research.

**Method:**

A qualitative focus group interview study was conducted of 47 adults (Marfan syndrome (MFS) = 14, Loeys-Dietz syndrome (LDS) = 11, vascular Ehlers Danlos syndrome (EDS) = 11, relatives = 11). The interviews were digitally recorded and transcribed verbatim. Significant themes were identified, extracted, and organised undergoing content analyses.

**Results:**

The two main themes and 10 subthemes identified; I. Psychosocial well-being; (i) Social engagement and activity, (ii) Self-sufficient in daily living, (iii) Participation in education and work life, (iv) Coping with fear related to the disease, (v) Being able to control and accept fatigue and pain, (vi) Maintaining active engagement with family and friends (vii) Finding health-promoting physical activities. II. Monitoring and meetings with the health service: (viii) Feeling safe and receiving coordinated care, (ix) Being recognized, seen, and accepted, (x) Receiving factual and sober information and advice. The sub-themes seemed mutually interrelated in terms of barriers, strategies, and facilitators for improving quality of life. There was high degree of consensus regarding the factors emphasized as important for QoL among the various diagnostic groups and the relatives.

**Conclusion:**

Based on our findings, to improve QoL in patients with sHTAD we should more effectively integrate the patient`s perspectives and voice on the elements crucial to QoL. In addition, it is vital for developing and customizing validated questionnaires to accurately reflect the factors deemed significant by this specific patient cohort. The research is limited on patients’ perspectives on QoL, and more research is warranted. This might also be crucial for identifying relevant validated QoL instruments that reflect the patients` perceptions of what is vital for QoL.

**Supplementary Information:**

The online version contains supplementary material available at 10.1186/s13023-024-03485-3.

## Introduction

### Heritable thoracic aortic disease

Hereditary Thoracic Aortic Disease (HTAD) encompasses a broad spectrum of disorders primarily characterized by aortic events, predominantly aneurysms or dissections [1]. HTAD is classified as nonsyndromic (nsHTAD) when confined to the aorta, and syndromic (sHTAD) when associated with extra-aortic features [1–3]. Genetic testing is essential since it allows confirmation of the etiological diagnoses for HTAD. In several papers an extensive list of human genes and other clinical features associated with HTAD is mentioned [4–6]. In a study of Renard et al. [4] approximately 53 candidate genes were described associated with HTADs, but only 11 genes (COL3A1, FBN1, SMAD3, TGFB2, TGFBR1, TGFBR2, ACTA2, MYH11, MYLK, LOX and PRKG1) were identified as “HTAD” genes as they were assessed as having a “definitive” and “strong” gene-disease association during the curation process [5, 6]. Mutations in the five last genes described above are known as nsHTAD, as they are associated only with vascular manifestations [4–6]. Mutations in the six first genes are known to cause sHTAD with systemic manifestations and genetic phenotype, including cardiovascular, craniofacial, musculoskeletal and ocular systems, and cutaneous features [4–7]. The most prevalent conditions of sHTAD are Marfan syndrome (MFS), Loeys-Dietz syndrome (LDS) and vascular Ehlers-Danlos syndrome (vEDS) [2, 6, 7]. The focus of the present study is sHTAD.

The most serious complications in sHTAD are related to the risk of aneurysm and dissection of the aorta and other large arteries and leads to life-long medical treatment. [2]. Life-threatening complications can require emergency intervention, with an increased risk of morbidity and mortality [2, 8]. Because of the risk of aortic dissection, many patients are advised to refrain from contact sports, to limit their physical exertion, and to control their blood pressure strictly [9, 10]. Unfortunately, this may lead to a sedentary lifestyle [9, 11–13]. Many have skeletal signs with hypermobile joints, chest deformities, and scoliosis [4–6]. Physical impairment, chronic pain, and fatigue associated with sHTAD may be exacerbated by the fact that most have no effective treatment or cure [2, 4, 14]. Living with a sHTAD may be vastly more complex than just its medical features [15–19]. Many aspects of an individual`s life may be affected such as family life, education, work-life, leisure activities, and quality of life (QoL) [13, 19, 20].

### Rationale for the study in the context of what is already known

QoL has in the recent years, increasingly been studied and recognized as an important element in clinical decision-making in genetic disorders [19, 21, 22]. Although health providers endeavour to enhance the well-being of their patients, a challenge arises from the fact that the term QoL encompasses a range of conceptually distinct notions [19, 23–25]. QoL constitutes a multidimensional construct that is inherently individual and varies over time, encompassing an integration of physical, emotional, and social dimensions. Authors often do not explicitly define QoL, but rather imply its meaning by the constructs measured [19, 21]. Other similar terms used in the literature of QoL are «satisfaction with life», «well-being», «life-satisfaction», «life happiness» and «Health-Related Quality of life (HRQoL) [19, 26–29]. Notwithstanding the absence of a uniform definition of QoL, it is widely accepted that QoL can be defined as an individual’s perception of their position in life, within the context of the culture in which they reside, and in relation to their goals, expectations, standards, and concerns [30]. Furthermore, HRQoL pertains to the aspects of QoL that are directly influenced by an individual`s health. It is commonly regarded as encapsulating the effect of both disease and treatment on disability and overall QoL [19, 30].

A plethora of diverse and divergent scales, both generic and disease-specific, have been devised to assess (HR)QoL. However, to the best of our knowledge, no disease-specific scales have yet been developed to specifically measure QoL in the context of sHTAD.

Our previously published systematic review concerning QoL [19] in people with sHTAD (MFS, LDS and vEDS), published in 2019 revealed that research about QoL within these patient populations is exceedingly scarce. Our systematic review disclosed that the majority of the studies focused on MFS, and very few studies dealt with other sHTAD, such as LDS and vEDS. Furthermore, the conducted research predominantly employed quantitative methodologies, utilizing a questionnaire for data collection, with the Short Form-36 (SF-36) being the most frequently used instrument for assessing (HR)QoL. No studies have been identified using a qualitative approach for investigating QoL in adults with sHTAD. To ensure up-to-date information, we conducted an updated systematic search for relevant literature on December 12, 2023, and identified seven new articles dealing with QoL in sHTAD since 2019. Only one of these seven new articles dealt with vEDS and LDS, while the others dealt with MFS, and no qualitative articles about QoL in adults with sHTAD were identified.

We propose that to effectively integrate patients` subjective interpretations of QoL determinants, it is crucial to consider their unique perspectives and narratives. Consequently, a qualitative methodology is necessitated to meticulously investigate and accord due weight to the patients` perceptions and meanings. It is anticipated that the findings of this study will enhance the understanding and provide a basis for the development of appropriate clinical interventions and management plans for this patient group.

### The aim and research questions

The main aim was to investigate the perceptions and opinions of what constitutes important determinants for QoL in individuals with sHTAD, utilizing a qualitative study approach. Further to discuss clinical implications and direction for research. The following research questions were employed: i) What do the patients and relatives emphasize as important determinants for QoL, ii) How can this be comprehended and understood within the concept of sHTAD and iii) What implications on clinical practice and further research does this have for the measurement of QoL within this specific patient cohort?

## Design, method and materials

### Ethical considerations

This paper is part of a comprehensive study of psychosocial aspects, physical function, and QoL in adults with sHTAD. The study has been approved by the Regional Committee for Medical Research Ethics (Health Region South–East) (2017/745).

All participants received both written and oral information about the study, and each participant was required to sign an informed consent form before volunteering to participate. The study was conducted in cooperation with the patient associations. A reference group, comprising two adults with sHTAD and one adult relative have been involved in all stages of the research process to enhance the quality and relevance of the study.

### Study design

A qualitative exploratory study of QoL in adults with sHTAD, including focus group interview methodology was utilized. The study was conducted in accordance with the COREQ-checklist for qualitative research [31]. Moreover, a phenomenological approach, informed by Heidegger`s philosophical constructs, was adopted [32]. This aligned with the study`s primary objective, which focuses on understanding the perceptions of QoL of people with sHTAD and their relatives. Focus group interviews were employed for the dual purposes of idea sharing and generation of knowledge [33]. The intention was to delve deeper into how individuals with sHTAD and their relatives articulate and reconcile their thoughts, feelings, perceptions, and interpretations of QoL. Focus groups have the potential to foster a more authentic and dynamic dialogue among participants, thereby facilitating a clearer elucidation of key insights [33, 34].

### Informants

Patients (≥ 18 y) with MFS, LDS and vEDS and other sHTAD registered at TRS National Resource Centre for Rare Disorders in Norway were eligible and invited to participate in the study. All participants had a medically verified sHTAD disease, diagnosed by medical experts (including genetics, thoracic surgeon, or cardiologist) and thereby referred to our resource centre. Patients with LDS and vEDS had a molecularly verified diagnosis, while patients with MFS fulfilled the Ghent criteria [35]. Medical records of all participants were reviewed to confirm the diagnosis. The exclusion criteria were: i) uncertain diagnosis ii) inability to speak/understand Norwegian language, and iii) serious psychological disease that interfered with study participation. The relatives were either spouses/partners or parents of someone with sHTAD diagnoses.

### Selection methodology

Sampling employed a hybrid strategy of convenience and purposive methods. To augment the study`s trustworthiness and ensure a diverse representation of individuals with different sHTAD, two distinct recruiting locations were selected through selection triangulation, designed group A and group B. In addition, the relatives in group C were included. Totally 47 people participated in this study.

In October 2017, a three-day workshop for LDS and vEDS was convened in Norway. Of 24 attending adults 22 (LDS = 11, vEDS = 11) consented to participate in the focus group interviews (Group A). Subsequently, in April 2018, a purposively selected sample of approximately 70 adults with MFS, attendees of the Norwegian Marfan Foundation meeting, were invited to participate in focus group interviews. Invitations were disseminated in advance, through advertisements preceding the meeting. The initial 14 volunteers with MFS who responded were included in the study (group B). In addition, 11 relatives participated (group C). The recruitment process, selection methods, and characteristics of the participants are illustrated in more detail in Supplementary Figure 1.

The objective was to encompass 30 to 36 patients and 10 to 12 relatives, a range deemed sufficient to capture a unique variation, identify common patterns, and achieve data saturation. We estimated that approximately 8 focus group interviews, with two focus groups allocated for each diagnostic stratum, would likely satisfy our saturation criteria. A comprehensive description of the selection methodology and the demographic details of the participants are delineated in Supplementary Figure 1.

### Procedures

#### Interview guide

A semi-structured interview guide was devised to structure the focus group discussion. This guide comprised open–ended questions derived from pertinent research literature [15, 19, 36, 37] and our clinical insight. An auxiliary interview guide, featuring more specific questions was employed to elicit detailed responses on particular topics. Prior to deployment, the interview guides underwent evaluation by the reference group and seasoned clinical researchers. This review process did not necessitate any modifications.

#### Implementation of focus groups

Focus group interviews were facilitated by pairs of experienced professionals, serving as moderators and co-moderators. All facilitators possessed extensive clinical experiences with sHTAD patients and had undertaken a specialized in-house training course designed for these specific interviews, in addition to being adept in the requisite interview techniques. Further elaboration on the study process is provided in Supplementary Table 1.

The interview typically spanned 110 min (ranging from 90 to 125 min). Moderators primarily directed the discussion, while co-moderators documented observations. Reflective journals maintained by the co-moderators provided additional insight into the process.

Post-interview, moderators convened in reflective sessions to distil key themes from the discussions. These preliminary themes were subsequently validated with the focus group participants, resulting in confirmation rather than amendments. Combined with the interview guide and relevant literature, these themes constituted a preliminary thematic framework for subsequent analysis. All eight focus group interviews were audio-recorded and transcribed verbatim by two independent researchers (GV, HJ), then anonymized and coded with distinct colours and pseudonyms for each participant.

### Analysis

The study employed an Inductive Systematic Text Condensation (ISTC) analysis, adhering to the principles outlined by Malterud [38]. This approach, rooted in psychological analyses, is particularly recommended for interpreting focus group interviews [38]. ISTC is advantageous when existing knowledge about the phenomenon is limited and does not necessitate a foundational theoretical framework. The analysis proceeded through meticulous, step-by-step process: (i) Overview: the interviews were read comprehensively several times to grasp the entirety of the content. (ii) Coding: meaning units within the text were identified, highlighting the core message and assigning a thematic code. (iii) Condensation: These coded units were then organized, abstracted, and condensed into cohesive themes and categories. (iv) Synthesizing: Subsequently, the fundamental meanings within the categories were interconnected to construct overarching themes and narratives [38, 39]. The analytical process was initially conducted independently by two researchers (GV/HJ), with their findings subsequently compared. Any discrepancies were reconciled through discussion, ensuring consensus was achieved iteratively. Additionally, the emerging results were deliberately discussed with other co-authors (GA, AØG, KFM) and the reference group members to enhance the study`s credibility [38]. The detailed steps of the ISTC analysis are elucidated in Table 1.

**Table 1:** Inductive systematic text condensation analysis, approximately here

Steps of ISTC	Means of establishing trustworthiness
Step 1: Overview	Achieving an overview of the data material by reading the text several times
Three Authors (GV/HJ/GA) transcribed the material and then independently conducted a thorough review of the content	Bracket out preconception with sharp awareness for the participants voice
Step 2: Coding	Looking for preliminary themes
Three authors (GV/HJ/GA) independently analysed the material, searching primary patterns and codes	Researchers discussed tentative themes
Step 3: Condensation	Systematic reviewing the text line by line to identify meaning units
Three authors (GV/HJ/GA) condensed the material independently, critically evaluating relevant themes and codes before involving the two other authors (KFM/AØG) in the process	Use of preliminary themes framework and tentative themes
Step 4 Synthesizing	Identify and organize data elements according to research questions
All authors recontextualized and collaboratively identified terminologies for main themes and sub-themes. The authors discussed the themes collectively and reached consensus	Coding-identifying, classifying and sorting meaning units into categorises and themes
	The categories were sorted, abstracted, and condensed into themes with internal homogeneity
	The themes and categories were discussed among the researchers
	Discussed with co-researchers and consensus on themes and themes naming
	Reconceptualizing researchers putting pieces of text together again
	Present analytic text with the most salient content meaning
	Analytic distance and discussion
	The underlying meaning in the themes and categories were linked together to create the main themes

### The utilization and translation of quotations

Extensive use of quotations is categorized under various themes and subthemes, accompanied by descriptions of our interpretations of the quotations, which are employed to illustrate and maintain transparency in the analytic process. This is demonstrated in Supplementary Table 3. Each quotation is translated from Norwegian using an English-native-speaking translator and have been discussed with the research groups to ensure that the quality of the Norwegian everyday language is equivalently articulated in English. We believe that this approach enhances the validity and quality of the included quotations and the analytical process.

## Results

### The informants

A total of 47 individuals were included in the focus group interviews; MFS (*n* = 14), LDS (*n* = 11), vEDS (*n* = 11), and relatives (*n* = 11). The participants with sHTAD represented different diagnoses, gender, age, education, and social backgrounds. Characteristics of the participants and the relatives are described in Table 2.

**Table 2:** Characteristics of the participants with sHTAD, approximately here

	Mean (SD)	Range
Demographic aspects		
Age, mean range	48	22–71
Women, *n*/%	20 (56%)	
Educational level (highest finished education) ≥ 13 years, *n*/%	20 (56%)	
Working full time (100%), *n*/%	14 (39%)	
Working part time combined with disability pension, *n*/%	5 (15%)	
Full time disability pension (100%), *n*/%	14 (39%)	
Students, *n*/%	3 (9%)	
Diagnoses		
MFS, *n*/%	14 (39, 0%)	
LDS, *n*/%	11 (30, 5%)	
vEDS, *n*/%	11 (30, 5%)	

Of the 11 relatives, 7 were female and 4 male. Six were both parents and spouse to people with sHTAD, 2 were only parents and 3 only spouse, and 8 worked full time and 3 were retired/disability pension. More detailed descriptions of the patients and their relatives are reported in Supplementary Figure 1.

### Themes and categories

**Fig. 1:**
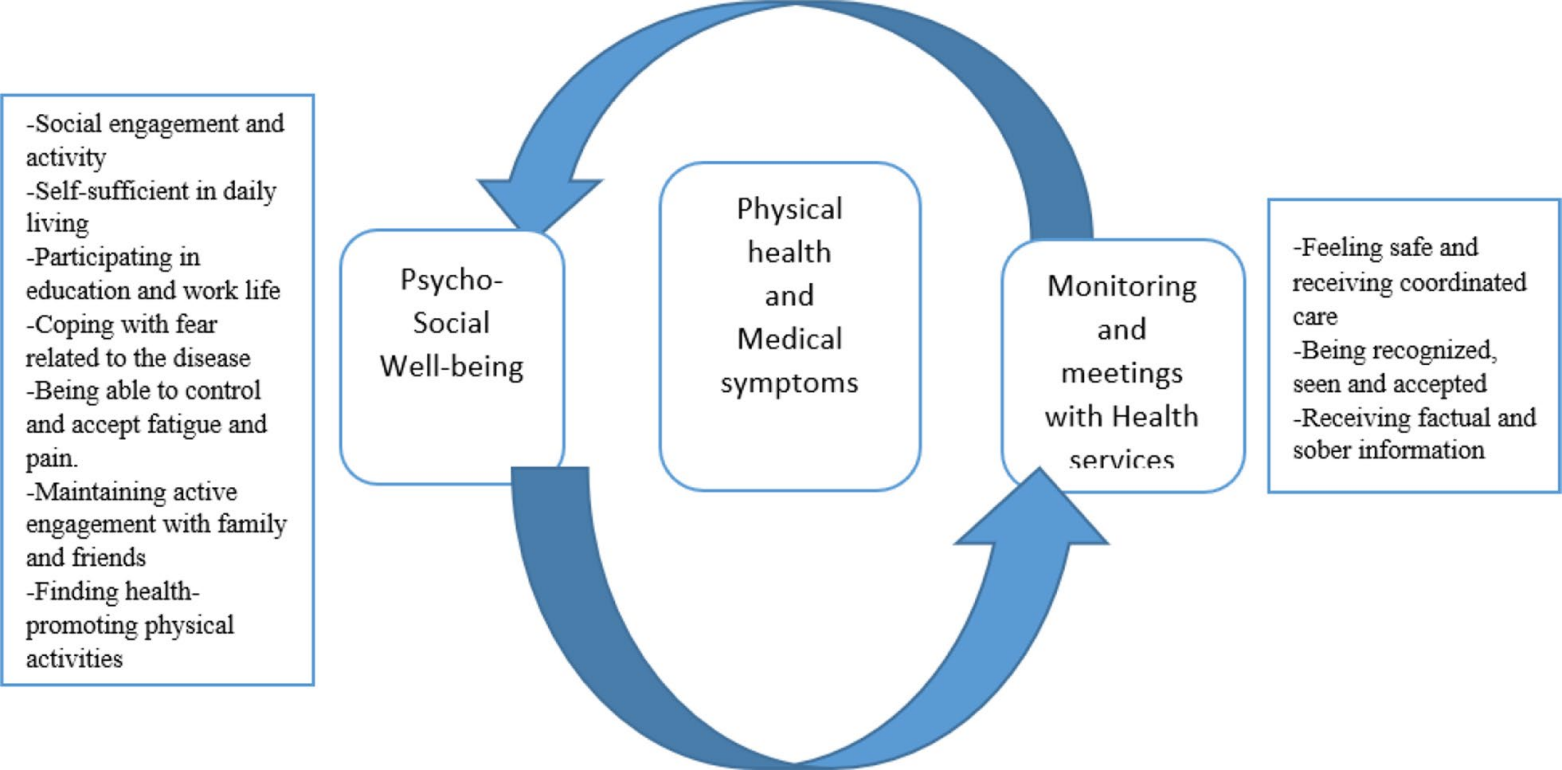
The main themes and sub-themes emphasized by the participants

Two main themes were identified as relevant to QoL (i). Psychosocial well-being, and (ii) Monitoring and meetings with the health service. Figure 1 illustrates the two main themes, and 10 sub-themes. An overall theme indicating that “being able to maintain independence while simultaneously receiving support and care” was emphasized as important, both from the patients and relatives.

Both patients and relatives were concerned with the potential medical life-threatening aspects of the disease, indicating that these aspects had central and decisive roles for QoL. Most of the patients also described that pain and fatigue had a great impact on daily function.

Overall, the material revealed that the majority emphasized the importance of identifying robust strategies to accept and manage the symptoms associated with the diagnosis, given the absence of any effective treatment regimen to cure the symptoms. This may have been the primary reason that the participants emphasized the psychosocial ramifications and medical follow-up provided by the health care system as critical determinants for QoL. The main themes, subthemes including the participants’ quotations and the interpretations analyses are illustrated in Supplementary Table 1.

#### Psychosocial wellbeing

The various sub-themes in the main theme are overlapping, rendering their delineation challenging, as they are extensively mutually interconnected. Nevertheless, we opted to categorize them into distinct sub-themes to elucidate the significance of each on QoL for both patients and relatives.

*Social engagement and activity*

Participants described the substantial potential to isolate and withdraw from all social activities, particularly during periods related to severe surgeries, as well as times of acute pain and fatigue. The relatives expressed feelings of exhaustion and uncertainty from continually managing an unpredictable situation. Consequently, many asserted the paramount importance of these interactions, as they provided not only increased support from their surroundings but also opportunities to engage in diversions from their illness. Both the relatives and patients reported that such engagement mitigated feelings of depression, reduced sensations of isolation, and imbued their lives with meaning.

*-I need to stay active and feel engaged, or life feels empty and meaningless. I can`t focus on the illness all the time, so I actually feel better if I can also support others; it gives me a lot* (Woman with LDS).

*-I just have to live life. It`s easy to become isolated, so I try to stay active. We travel quite a bit around the world, it`s important for me. You only live once, and you can`t let the illness hold you back. I don`t want to spend all my time worrying about what could go wrong; that would lead to a very narrow life, and I don`t want that (*Women with vEDS).

*Self-sufficient in daily living*

The significance of self-sufficiency was emphasized, despite that most reported managing activity in daily life. However, some noted that they occasionally required assistance with more strenuous tasks such as vacuuming and snow removal. Some families also described a kind of strategic division of labour to ensure that individuals with the condition are spared from the more arduous tasks. The interviews indicated that self-sufficiency was not just about the physical aspects of handling daily tasks; it`s deeply intertwined with dignity, autonomy, and an individual`s ability to feel integrated life within society.

-*Yes, when you start wondering what the possibilities are, you begin to see them – I really want to do everything, and I don`t feel limited, so I can do carpentry, paint or mow the lawn. This is important for me, even though sometimes I have to ask for help, which I don`t particularly like* (Man with LDS*).*

*-I used to be the one who fixed everything, managed everything. But it`s not like that anymore, and it`s tough to accept. It`s a sorrow to feel this decline and ask for help. Being self-sufficient in daily living is important for my quality of life* (Woman with MFS).

*Participating in education and work-life*

Education and work were reported as important for numerous reasons, significantly impacting their personal development, economic status, and social integration. Some described being advised to avoid physically demanding and strenuous work, hence a number expressed that they had opted for theoretical education, while others recounted the necessity to adjust post-diagnosis, transitioning from masonry or carpenter to more sedentary work. These transitions could be burdensome for some, as a significant portion of their identity and self-esteem were tied to their previous job position. For some, the diagnosis had significant implications for both career and occupation readjustment. The majority emphasized participation in work was central for QoL, even though several asserted that they had managed to adapt and found meaning in life even while being on disability benefits. Work was also described to reduce the feeling of isolation and increase the feeling of competence and self-worth. Others claimed that engagement in education and work could provide a sense of purpose, routine, and structure.

*-Education was important for me, especially when I couldn`t do heavy work. So I`ve become much more interested in theoretical issues; it`s good for me (*Young man with vEDS).

*-We are both currently working full-time, so we`re fortunate in that regard. However, I was really worried because my husband has always worked as a bricklayer, and he had to quit. It wasn`t easy for him to find job alternatives that matches his skills and abilities, but eventually, with some great help from the social services, he made it work* (Woman relative)*.*

*Coping with fear related to the disease*

Coping with fear related to the disease seems a deeply personal and ongoing process, both for the patients and the relatives, and the strategies seem to change over time. For some the consequences of fear may lead to avoidance (ignoring symptoms) or hypervigilance (excessive worry about their health). Both strategies might be detrimental. Some also described that fear sometimes caused them to withdraw and isolate themselves. While others described that, they had found more effective coping strategies that helped them maintain and enhance social support networks, which were emphasized as crucial for emotional support and practical assistance. Some also described that connecting others in the same situations or through patient organizations could provide comfort, understanding, and practical tips for coping.

*-It is not so easy, every day I listen to my heart, to my body - if it tells me something is wrong, sometimes it`s very exhausting, and at times, I find it difficult to be with others when I worry so much* (Man with vEDS).

*-I feel that having contact with those who share my diagnosis is beneficial. I`m involved in the patient organization, and it`s important for my; not only can I offer my support, but I also receive a lot of support in return* (Woman with MFS)*.*

*Being able to accept and control fatigue and pain*

Most of the patients reported experiencing intermittent pain and fatigue, describing these symptoms as significant impediments in daily life. Some claim that it is about not letting these symptoms define their existence. They tried to find ways to coexist with them, minimizing their impact, and focusing on what brings joy and fulfilment. Others described that they needed to learn how to balance activity and rest to prevent overexertion in managing these symptoms. Both the patients and the relatives emphasized the importance of understanding the causes and mechanics of these symptoms because they seem so unpredictable and often appear without an apparent cause.

*-It`s tough dealing with both pain and fatigue, and sometimes they really impact my life. But I do my best to push them aside. I try not to let them dictate me, and I`m stiving to live my life with them simply tagging along* (Woman with MFS).

*- I react so differently, that`s a problem. While others improve with activity, for me, a long walk is just exhausting. Afterwards, I feel drained, zoned out and not fully present. This might be another diagnosis, such as fatigue or chronic fatigue syndrome, so it`s challenging to discern what`s what and how to proceed* (Women with LDS).

*Maintaining active engagement with family and friends*

Several emphasized that maintaining active engagement with family and friends is important for maintaining social networks and support and for some this seems vital for coping with daily challenges both for those with the disease but also their relatives. Some described relations with families as a way of providing a sense of normality and an antidote to stress and anxiety. Others reported that having a family and good friend fosters a sense of belonging and can help navigate life more effectively and joyfully. Being a relative to someone with a potentially life-threatening condition can be extremely challenging, yet for some, it also fosters a more intense sense of togetherness and mutual support. It was also mentioned that family and friends can offer practical help, whether it`s assisting with daily tasks, navigating health care, or providing transport.

*-Sometimes, I feel like she`s a ticking bomb and I’m anxious all the time. It’s important for us to be able to talk about the challenges we face together, while also taking good care of each other to provide the necessary support in our daily life* (Male relative).

*-My family and friends mean a lot to me; I couldn`t do it without them. They make me laugh and see the brighter side of life. But sometimes, it`s my turn to lift them up and make life bright and fun, so it`s not just worry and sadness that consume us. Otherwise, there wouldn’t be much of a life* (Female with vEDS).

*Finding health-promoting physical activities*

Finding health-promoting physical activities seems crucial for people with sHTAD and their relatives. It seemed like a key component of managing their condition and enhancing their overall health and well-being. Most of the participants claimed that they had received information and advice on activities with low impact and moderate intensity, but still, several claimed that they encountered difficulties in practical application. For most patients and relatives, regular check-ups and monitoring, in addition to consulting healthcare providers to understand which activities are safe seems crucial to ensure that the physical activity is not adversely affecting their health. Some described that successful engagement in physical activity is important for giving a sense of control and accomplishment.

*-Yes, the advice on restrictions is sometimes very confusing. I was told that I should try to avoid overexerting myself, but still engage in some activities. However, there`s no clear guidance. I wish someone could provide concrete advice, so I could regain a sense of control and motivation for physical activity (*Young male with vEDS).

*-The advice and recommendations regarding physical restriction are crucial, and I always inquire about which activities I can engage in. It`s important for me to stay active and participate in physical activities* (Man with MFS).

#### Monitoring and meetings with health services

The patients and relatives claimed that they are highly dependent on health care, as people with sHTAD require lifelong regular follow-up particularly concerning the aorta and other severe symptoms of the disease. This may be the reason why the participants emphasized that they are highly reliant on their connection with the healthcare system and that this greatly impacts their QoL. Three sub-themes emerged as important determinants for QoL related to the support system.

*Feeling safe and receiving coordinated care*

The interviews indicated that feeling safe and receiving coordinated care was not just comfort; it was described as a fundamental need that significantly impacts the psychological and physical journey of the patients and their relatives. sHTAD affect multiple body systems, including cardiovascular, skeletal, and ocular system, and coordinated care was described as important for ensuring all aspects of the condition were addressed comprehensively. They also emphasized that feeling safe and cared for is important for building trust between them as patients and their relatives with healthcare providers. Trust seemed also crucial for effective communication and collaboration in care management.

*-Sometimes I feel reassured that they have the time and dedication to take care of my health, alleviating some of my worries. Then I feel I can trust them and then it is easier to talk about all my questions (*Woman with vEDS).

*-It`s burdensome when I don’t feel like I`m receiving the follow-up care I need from the hospital and keep encountering new doctors who aren`t familiar with my diagnosis. That`s a bit stressful and affects my inner self. We`re not supposed to have any stress, so then it becomes even more stressful.* (Man with vEDS).

*Being recognized, seen, and accepted*

Recognition and acceptance from healthcare providers were emphasized as important for emotional support, reducing feelings of isolation, and for some being seen and taken seriously was described as improving the mental well-being. Some also claimed that when they felt understood and supported, they were more likely to communicate openly about their symptoms, concerns, and preferences.

*-I was very surprised by how often the surgeon who operated on my husband spoke with me It gave me a feeling that he truly saw me – it surprised me and was very positive. I genuinely felt I could trust him* (Female relative).

*-I feel they see me for who I really am, understanding my needs and problems, especially when they acknowledge my fear, fatigue, and pain. When they understand, I don`t feel so alone anymore* (Man with LDS*).*

*Receiving factual and sober information*

Providing factual and sober information and advice seems vital for effective disease management, building trust, and ensuring compliance with treatment for these patients. Patients and their relatives articulated considerable uncertainty and anxiety related to prognosis, management of daily symptoms, and the handling of received information. They express a substantial dependence on the reliability of advice from healthcare professionals. Therefore, the interview suggests that accurate and factual information, as well as concrete and practical guidance in daily life, holds exceptional value for this group. This may help them to actively participate in their healthcare and set realistic expectations. Despite, that truth may sometimes be difficult to hear, some claim that uncertainty and misinformation are worst because it can lead to increased anxiety and fear. Clear, factual information may alleviate unnecessary worries and allow patients and families to focus on coping and treatment.

*-It`s easier to follow the doctor`s instructions when the information is clear, practical, and easy to understand. That way, I can apply it effectively in the real world* (Woman with vEDS).

*-When I have received relevant knowledge and I can trust the doctors, their advice seems reasonable, while they listen to me, I feel we can cooperate in a better way, I believe this is best for my health* (Man with LDS).

## Discussion

To our knowledge, this is the first qualitative study of QoL among adults diagnosed with sHTAD (MFS, LDS, or vEDS). Overall, there are several factors that can significantly reduce or improve the QoL. These factors include disease-related variables, as well as a large number of psychosocial factors. The findings indicate that the various health aspects of the sHTAD diagnoses critically impose constraints on life activities. When medical symptoms directly cause health issues, the psychosocial response to these symptoms and the nature of the support system significantly influence the perceived QoL for both patients and relatives.

### Psychosocial well-being

The seven subcategories of psychosocial aspects exhibit considerable overlap; nonetheless, participants underscore unique considerations within each category. The QoL perceptions are predominately centred on social engagement, maintaining self-sufficiency in daily life, family life, and the pursuit of educational and vocational opportunities. Furthermore, there is a significant focus on mastering challenges associated with the fear emanating from the potentially life-threatening nature of the diagnosis. Additionally, a substantial emphasis is placed on developing acceptance and effective coping strategies for managing pain and fatigue, alongside navigating health-promoting physical activities. Each factor was identified as pivotal for QoL.

Other studies have found that the perception of the disease has substantial impact on how people cope with the disease and their perception of QoL [19, 20, 25, 40–43]. In our study, most participants described the need for support for managing the fear associated with potential cardiovascular complications, yet the cardiovascular manifestation may be underestimated as long as the individual experiences no subjective complaints [19, 25]. Concurrently, our research indicated the imperative of recognizing the palpable apprehension of aortic dissection or other serious medical complications for both patients and relatives. However, this perception is not necessarily correlated with the medical severity of the condition [13, 19, 25, 37, 44, 45, 49]. It appears that the subjective severity and its consequential impact on QoL predominately stem from the manifestations deemed by patients and their relatives to induce disability. Notably, symptoms like pain and fatigue are perceived to pose substantial challenges in daily life [12, 14, 16, 17, 19, 20, 25, 46]. Despite that many in our study described that pain, fatigue, and fear of aortic dissection were the most challenging symptoms to deal with in daily life, they experienced that healthcare personnel avoided talking about these ailments, and only focused on the objective measurable criteria of the condition. The difference between the medical severity and subjective severity suggests that patients perceive the disorders differently from professionals. This is crucial for healthcare providers to acknowledge when discussing patient-reported symptoms and their potential impact of QoL in clinical practice.

Moreover, this can also be viewed in relation to that several patients and relatives considered the positive effect of features such as optimism, positive thinking, and hopefulness in the disease acceptance process and believed that enjoying such features would favour the process of adapting to the disease. Studies show that optimism and positive beliefs, and the possibility of participating in society in general are significantly and positively correlated with different aspects of QoL, and being optimistic and having positive beliefs play a key role in preventing depression and fostering hope [47, 48]. Some claimed that family life and friends, the possibility to talk to others in similar situations, and contact with the patient organizations were factors that may increase positive thinking.

Several participants also emphasized that participation in education and work is not only associated with economic or social benefits, but is also vital components for dignity, self-efficacy, and Qol, similar to those found in other studies of patients with sHTAD [19, 37, 49–52]. Patients with sHTAD likely face few physical restrictions in education and employment, aside from avoiding heavy lifting and strenuous physical exertion. Empirical evidence suggests a propensity for high educational attainment and successful occupation integration among this cohort [37, 49, 51]. Nonetheless, concurrent studies have illuminated the prevalent challenges they face, notably in managing persistent fatigue and pain, which can impede their educational and professional endeavours [37, 49, 51]. Recognizing these impediments, it is imperative for healthcare and social service professionals to acknowledge and address these issues proactively. Early and targeted support is crucial to mitigate the risk of educational and occupation exclusion, thereby enhancing their QoL and societal contribution.

A final sub-theme emphasized by the participants as crucial for QoL was the identification of effective strategies for health-promoting physical activity. Although the majority had received advice regarding restrictions related to physical exertion, many expressed a lack of comprehensive understanding about the types of physical activities they could safely engage in, similar to those found in other studies of these patient groups [9, 11, 12]. Maintaining fitness was a priority for most, yet the conversion of these guidelines into practical, safe routines proved to be a source of frustration and difficulty for many, similar to those found in other studies of physical activity in these patient groups [11, 12, 53]. Physical activity is particular important for this groups, as a recently published longitudinal study [54] of adults with MFS found that after 10 years, older age predicted a decline in physical health-related QoL (physical function and bodily pain), while the mental domain remained unchanged. This study also emphasized support measures to prevent physical decline and not only focus on organ pathology in the follow-up of patients with MFS. The participants in our study expressed a desire for more detailed, personalized guidance to navigate the balance between beneficial exercise and their health limitations similar to found in other studies of patients with sHTAD [12, 53]. This indicates that healthcare professionals need to emphasize the importance of regular exercise, and potentially recommend activities that are safe and suitable for these patients.

### Communication and relation with Health care

Several studies have reported that people with sHTAD depend on support and assistance from medical professionals to deal with everyday problems that may induce disease and stress [12, 14, 55–58], but few studies have investigated the associations between QoL and contact with health services.

Our results indicate that the interaction with the healthcare services appears to play a central role for the participants, significantly impacting both the patient`s and relatives’ QoL.

Three nuanced subthemes emerged, exhibiting considerable interrelation, yet distinct in the elements participants delineate as pivotal to their QoL. Overall, feeling cared for and recognized, experience coordinated care, and receiving relevant factual information may substantially increase these patients’ well-being and QoL. Support from medical professionals seems highly important, not only in medical aspects but also in psychosocial aspects. Thereby indicating that healthcare professionals` engagement and the coordination of health services across various departments are critical facets for enhancing QoL and feeling more safe.

This is in contrast to studies of Shimizu et al. [56, 57] which found that accessible consultation did not affect the subjective well- being of people with MFS. They argued that individuals with MFS often ignored their diagnosis to forget the fear of sudden death. This is similarly found in a few participants in our study, however, most participants emphasized the importance of information and enhanced knowledge about their diagnosis. The studies of Shimizu et al. [56, 57], posited that even though participants could consult about treatment, they felt life was too complicated. Thus, preventing them from receiving pertinent advice and education for such difficulties and concerns from medical personnel. They concluded that even with medical consultations, patients did not receive appropriate advice and psychological support for the complex issues they faced [56, 57]. Our findings did not corroborate these results. This may be an indication that the healthcare systems are organized differently in Japan from Norway. Most participants in our study reported that interaction with healthcare professionals, the medical monitoring, and the advice they received were crucial for how they managed the disease in daily life and QoL.

Studies have found that feeling safe and cared for by healthcare professionals can provide emotional and physiological support, which seems essential for coping with the uncertainty and fear that often accompany such diseases [59]. Regular monitoring of medical symptoms and coordinated care seems to increase the feeling of control. Studies have also found that being recognized and accepted may reduce fear and empower patients and relatives to make informed decisions about their care [60, 61]. Our results indicate that healthcare professionals should not only support patients in solving problems themselves, but also promote family involvement in care [62]. For relatives, these aspects help them understand the patient`s condition, what they can expect, and how they can provide support. It also ensures that they are acknowledged as part of the care and support network, which is vital for the patients’ and their well-being. By promoting meaningful dialogue between family members, the patients’ self-perceived burden on the family can also be reduced [62, 63]. The medical staff could assess the potential for patients to manage the disease and guide them in understanding its potential benefits, thereby assisting them in making appropriate psychological adjustments. Studies have also found that accurate and concrete information is critical for patients and their families to make informed decisions about treatment options, its progression, and potential outcomes empowering them to be active participants in their care [64, 65]. When patients feel they are treated with respect and their concerns are taken seriously by their healthcare providers, this might be crucial for effective care and adherence to treatment plans.

### Clinical implications for healthcare providers

In summary, the sub-themes emphasized by the participants are not just important; they are integral to ensuring that living with a life-threatening condition is not just survival, but about maintaining dignity, understanding, and QoL both for the individuals affected and their families. The goal for the next frontier of healthcare for individuals living with sHTAD diagnoses may be to improve QoL, not only by advancement in medical treatment but also with interventions aimed at modifying psychosocial and contextual factors that influence QoL [19, 25]. Patient-reported QoL should be incorporated into clinical practice to ensure the patient`s and relatives’ perspectives are included in clinical decision making. Because a sHTAD condition affects every aspect of their daily life, interventions aimed at enhancing QoL by adjusting psychosocial factors need to be designed and tested [19, 25]. Intervention might aim to adjust the appraisal of the stress evoked by the threat of the condition.

### Direction for further research in sHTAD

There is a great need for further studies on sHTAD diagnoses that will aim to advance our understanding of QoL, both as a concept and as an important outcome. The importance of conceptual clarity, rigorous methodology with appropriate QoL scales, and theoretically.

grounded research must be emphasized. Then, the research will yield more evidence-based and relevant for clinical applications and interventions to facilitate improvement in healthcare and counselling. Given that only quantitative studies have been identified addressing QoL in adults with sHTAD, there exists a significant necessity for additional qualitative research. Understanding what patients and their relatives prioritize as important is central to capturing the subjective perceptions of QoL. This may give a more complete understanding of how the patients may perceive QoL in different contexts and which factors are important for increasing QoL. Nearly all studies of QoL are conducted in Western countries, the assessment of QoL in patients with sHTAD diagnoses should also be further explored in other cultures and geographical regions, including Eastern Europe, South America, Asia, Africa, and Australia. It is a challenge to conduct studies on rare disorders, because of small sample sizes. International collaborative studies, using the same study design and disease specific QoL measurements and only including people with clinically and genetically verified diagnoses are recommended. This might contribute to a better understanding of how the diagnoses and health symptoms may influence QoL, also across diverse cultural differences. The European countries could also collaborate on a joint project focusing on QoL in adults and children with sHTAD. In addition, to achieve better knowledge, international researchers can cooperate on developing a disease-specific scale for measuring QoL for patients with a genetic cardiovascular condition. Disease-specific measures would be more responsive and clinically useful and will give a more exact picture of how the diagnoses impact QoL across different cultures and contexts.

## Limitations and strength

There are some limitations related to this study, utilizing focus group interviews. The retrospective perspective of the participants, remembering experiences and events may imply a possibility for recall bias. Recruitment from the Patient Organization meeting and workshop may imply selection bias with the potential of recruiting motivated and interested participants willing to use time for focus group interviews. Therefore, our findings may be limited to the patients with sHTAD and relatives who were willing to talk about their experiences and challenges. Another limitation may be how the term QoL were interpreted by the participants. They may have different understandings of these concepts, but one of our intentions was also to examine the differences in perceptions and thoughts about the concept of QoL.

A strength in our study might be that both the moderators and the co-moderators were experienced clinicians and/or researchers, and they underlined their interest in all types of narratives. In the analysis, we carefully tried to identify and exclude repetitive patterns concerning our expectations and pre-understandings, as recommended in the literature [33, 34]. To enhance the validity of the study, we have endeavoured to maintain transparency by including a thorough overview of the analysis process in a Supplementary Table. This encompasses extensive quotations categorized within various themes and sub-themes, as well as a description of the basis on which the analysis was conducted. In addition, anonymized data are available on request to the authors (TRS, National resource Centre for Rare diseases).

## Conclusion

This study is the foremost qualitative investigation into the subjective perceptions and considerations of adults with sHTAD and their relatives concerning the determinants of QoL. The results illuminate that, although medical symptoms serve as the immediate etiological factors for health concerns, the ways in which individuals psychologically and socially react and the relations to the health care professionals exert a profound impact on their perceived QoL. As most individuals with sHTAD will not be cured in their lifetime, identifying ways to improve QoL is of utmost importance to patient-centred care.

The awareness of the facilitators illuminated in this study might help families, healthcare systems, supportive organizations, and society to increase the supportive educational and clinical service and ultimately improve QoL. Further research is required to better understand the potential importance of QoL, which will affect the organization and content of the assessment and management of adults, but also children with sHTAD.

## Supplementary Information

Supplementary file 1.

Supplementary file 2.

## Data Availability

The dataset (transcribed interviews) from the qualitative focus group interviews are available, but restrictions apply to the availability of these data, which are used under licence for the current study and are not publicly available. The data concerns a group of adults with sHTAD and some relatives. The qualitative focus group interviews are confidential and sensitive with personal data involved. The data, are however available on reasonable request to the corresponding author: (Gry.velvin@sunnaas.no).
