# Supplementary figures and images for: Quality of life in people with syndromic heritable thoracic aortic disease and their relatives: a qualitative interview based study
Source: Orphanet J Rare Dis. 2025 Jan 9;20:12. doi: 10.1186/s13023-024-03485-3 (PMC11714953; doi:10.1186/s13023-024-03485-3)

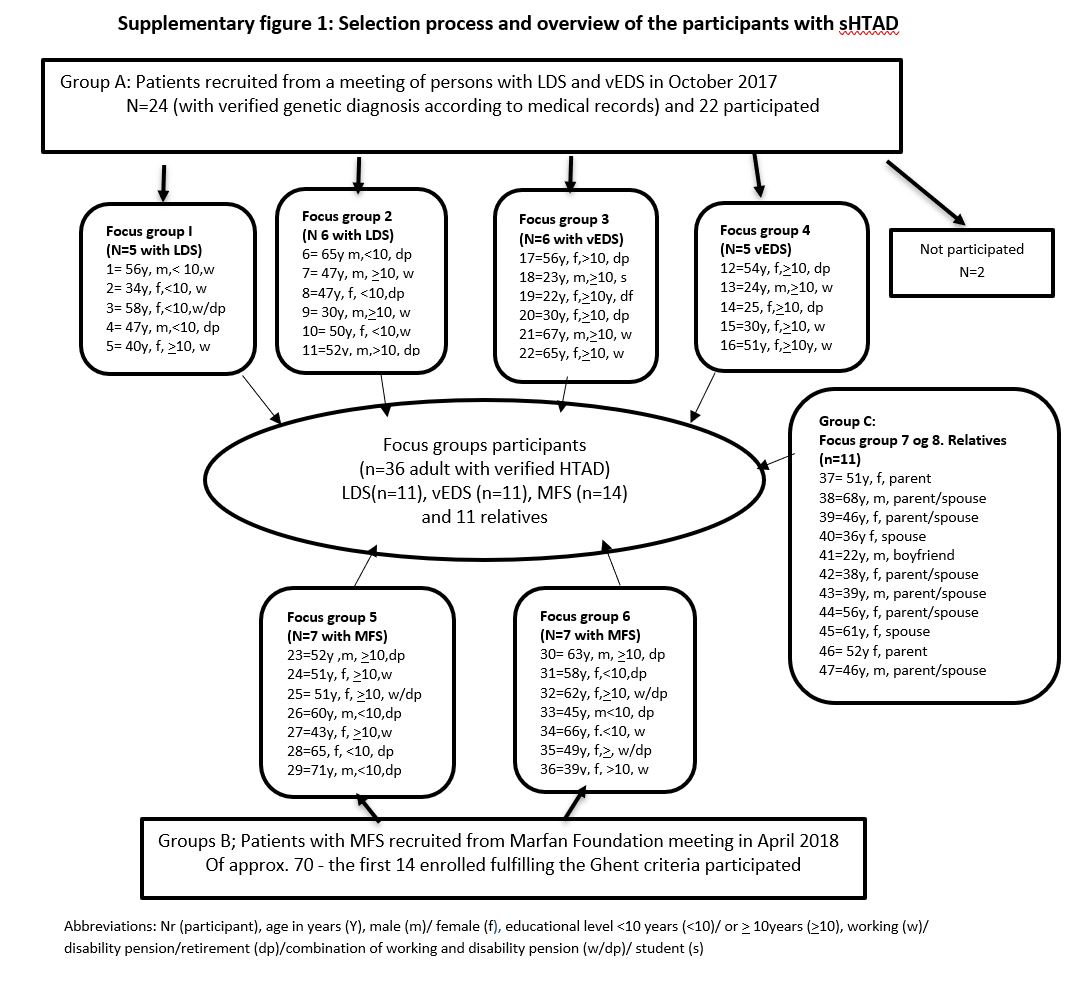

Supplement: Supplementary file 1 [file 13023_2024_3485_MOESM1_ESM.jpg]
