# Supplementary material for: Quality of life in people with syndromic heritable thoracic aortic disease and their relatives: a qualitative interview based study
Source: Orphanet J Rare Dis. 2025 Jan 9;20:12. doi: 10.1186/s13023-024-03485-3 (PMC11714953; doi:10.1186/s13023-024-03485-3)
Supplement: Supplementary file 2 [file 13023_2024_3485_MOESM2_ESM.docx]

| **Supplementary table 1** | | |
| --- | --- | --- |
| **Psychosocial well-being** | **Quotations** | **Interpretations of meaning** |
| Social engagement and activity | *I`m doing great, we`ll see how it goes, I decided this is going to be okay, and tried not to focus on the challenging parts, and I`ve been lucky, things are pretty well, after all. I is important for me to take part in activities and feeling alive (Yong man with LDS)*  *-I think it`s good to take that hit, at least for me. I go for it, and then I come home completely wiped out, and then, as I say, it`s important to have selective memory, forget it and just keep going, because then something happened, and I `d rather be exhausted for a few hours (Man with MFS).*  *-Yes, I like being active, and a shopping trip can give me lots of energy, a boost that I know I need. But then it`s like I can`t do some much more that day, but I like it (Woman with vEDS)*  *- With activities and stuff, and having the diagnosis, I don`t think about it anymore. As you live with the diagnosis, you learn to cope with it, but first, you go through the uncertainty, not doing anything, just boring stuff, with little to look forward to. At some point, I think we all understand that we have to try to do something, being alive and active helps (Man with MFS)*  *-For me, the diagnosis takes up a lot of time and space in life, thoughts around managing energy, what I should make room for, and what I shouldn`t, it`s not always so simple, I`d like to make room for most things (Man with vEDS)*  *-You have to share how much space the disease will get. It's all consuming, in its own way. That you have to learn to live with it without it taking up too much space, more space than necessary.(Man with vEDS)* | -It seems that social engagement and activity play a pivotal role in enhancing the physical, mental, and emotional well-being for people with HTAD, contributing to a more fulfilling and integrated life.  - Social engagement was emphasized as a key to integration and engagement in activities they can enjoy. It brings joy, satisfaction and a sense of achievement, and seems to improve QoL.  -Engagement in social activities seems also to enhance self-esteem and confidence and fostering a sense of empowerment.  -Social engagement and interaction with others can also boost mood, reduce feeling of isolation, and helps in fostering a sense of belonging and purpose. |
| -Self-sufficient in daily living | *-Yeah, when you ask yourself what possibilities are, then you see them – I really want to do everything, and I have no limitations, so I can do carpentry, paint mow the lawn. It`s a bit hard to understand when I hear you talk about limitations and low energy. I tend to think it`s laziness, but that`s because I don`t fully understand it. I tend to think it`s laziness, but that`s because I don`t fully understand it. Not everyone has the same symptoms and issues with these diagnosis, and I`ve been lucky. So I`m so pleased that I can be the one who helps others.(Man with MFS)*  *-I usually just set myself to autopilot, I feel the tiredness but avoid sitting down. Because I know if I sit, still be hard to get up. So, I always try to pace myself. There`s a lot of grief in this, as I wasn’t like this before. But for me, It`s vital for both body and soul to stay active. (Woman with vEDS)*  *-I don`t think I`m that different from others. I have some restrictions and limitations. And even though I feel I fall short, it`s okay. Because when I start to feel like I`m falling short, I stop exercising, then the depression hits, and everything quickly spirals down. And if I lie down on the couch then, the biggest exercise is getting back up again. It`s a lot about mentality- what I call “the threshold mile”. (Man with LDS)*  *-I used to be the one who fixed everything, managed everything. But it`s not like that anymore, and it`s tough to accept. It`s a grief to feel this decline, and ask for help. For me being self-sufficient in daily living is what you can call quality of life (Woman with MFS)*  *- I'm used to fending for myself, and then I know the limitations now, and it's such a small defeat for me, a personal defeat because I'm used to fending for myself, and then I actually have to ask for help to get things done, and I think it's terrible. (Woman with vEDS)*  *-Used to handling things on my own, so feeling these limitations, it`s like a personal defeat for me, because I`m used to being self-sufficient. Then, when I actually have to ask for help to get things done, I find it awful (Women with MFS)* | -Overall, the ability to navigate and perform daily tasks independently seems important for improving QoL.  -They emphasize that it allows for a more flexible, spontaneous lifestyle and opens up a wider range of activities and opportunities.  -Self-sufficiency was also emphasized to equips individual with the skills and confidence needed to adapt to changes and handle life`s challenges more effectively.  -It seems that self-sufficiency is not just about the physical aspects of handling daily tasks; it`s deeply intertwined with dignity, autonomy, and individual`s ability to lead a fulfilling and integrated life within the society. |
| Participate in education and work life | *- Education was important for me, particularly when I can`t do heavy work, so I`m much more interested in theoretical issues, that`s good food for me (Young man with vEDS)*  *-I`m student, and at the university I feel complete normal. I`m developing, learning a lot of new things, and like being together with the other student. I really feel that I`m good at it.(Young woman vEDS))*  *- I've become very good at prioritizing - because on the days I'm at work, I don't have any appointments in the evening, so work has taken the quota that day, you could say. So I'd rather do the housework and what to be done, when I have time off. So I'm not in the office every day, then, but rather have two days in the office and one day in the home office then, so then I have two full days off a week, as I do nothing, mmm (Man with MFS)*  *- At my job I haven't said I have Marfan. I haven't done so because I can't stand all the hundred questions or discussions, or when we sit at lunch and they have to analyse all the diseases, and give good advice and blah, blah. So I have completely distanced myself from the disease, and that I am completely normal and live a completely normal life. Off course, I have friends and family who know everything, but exactly at work I have such a free zone (Man with MFS).*  *-I had to ask for help from the municipality, because it was a bit much, but when I had to pay a lot of money for them to come and wash for 2 hours, I couldn't afford that - so then I just had to wash myself, but if there had been some such arrangements for us with such expenses it had been much easier (Male spouse to female with vEDS)*  *- Yes, now we are both in full-time work for the time being, so we are lucky that way, and I am grateful for that, that I am healthy, and you don't get healthier by thinking about bad finances either (Female spouse to male with vEDS)*  *- Yes, it has to be life in general then, then it has to be finances, finances are very important, you can't get by without money, and it's expensive to have bad health (Man with MFS).*  *-But I think the worst thing is that they said at that I wasn't allowed to work anymore, and it eats away financially, and I got disability benefits eventually, but it took time, and when the economy is significantly down, it is more intense than having a Marfan - it is felt in a different way (Man with MFS)*  *-For me work is much more than the financial, it is also important, but at work I feel confident and like to work with my colleagues. I really love my work, despite that it sometimes feels a bit heavy (Man with MFS).*  *-At work, they thought I was completely healthy after surgery, expecting me to come back fresh and rested, but that wasn`t the case (Man with MFS)*  *-No one at work really knew about my diagnosis, not until the dissection, then I had to tell. But for me, work has always been a place where I want to appear healthy and be like everyone else (Man with vEDS)* | -Participation in education and work seems important for numerous reasons, significantly impacting their personal development, economic status, and social integration.  -Education was important for personal growth, helping them to acquire knowledge, skills and confidence, and to explore their interest and realize their potential, leading to personal fulfilment.  -Participation in workforce seems to enable them to earn their own income, reducing dependence on family of social services. Many described it as crucial for self-esteem and security.  -Some claimed that engagement in education and work could provide a sense of purpose, routine, and structure. It can reduce feeling of isolation and helplessness and increase feeling of competence and self-worth.  -Many participant indicated that work is not just about economic necessity; it`s about respect, equality, and a feeling of contribution to society.. |
| -To cope with fear related to the disease | *-It not so easy, every day I listen to my heart, to my body - if it tells me something is wrong, sometimes this is very exhausting and sometimes I find I difficult to be together with others when I worry so much (Man with vEDS)*  *-I feel that having contact with the others with the same diagnosis as me, is good. I`m part of the patient organization, and that is important for my, both that I can support, but also receiving a lot of support from them (Woman with MFS)*  *-I think I was happier before, before I got rid of the ghost of a heart, yes, because it is not certain that I will have any heart problems in my life, but I always have to take it into account. The question of having children was very tough, so in a way I didn't get rid of it, but at the same time nice to know. (Woman with LDS).*  *-For me the big question was whether I should have children, a dilemma about daring to have children, both on my own behalf and on behalf of my children, Both the children who do not get Marfan, who might outlive the mother, if something were to go wrong, and the children who would inherit it. (Woman with MFS).*  *-The question of having kids was really tough, so in a way, I`ll never be rid of it, it haunts me wherever I go but at the same time, it was good to know (Women with vEDS)*  *-Yes, I think there are big changes after the operation, it was like completely different before, and especially the fact that I haven't returned to work, and it's noticeable, it's going to be different, so my job now is to go to a physiotherapist, and undergo 5 treatments a week (Man with LDS).*  *- He has been ill for a long time ... and we didn't know that before the operation, a few years ago, but even if we don't want it, I better follow that we know what he has. After he's operated, it's no longer like that dangerous that they know what we do" (Female spouse to adult with LDS)*  *- Being a relative is pretty much the same no matter what illness people have - and as a relative is a relative - and that presents a challenge, which I think is more or less the same, perhaps not in practical terms, but in purely emotional terms, the same issues arise (Female spouse to male with vEDS)*  *- It is clear that the helplessness is there, yes, that you would like to - with my husband then - who is in a lot of pain, and would like to help him that and cannot do anything to alleviate any of the chronic pain he has . But life in general, we have managed to adapt very well, so everyday life is completely normal for me. (Female spouse to male with vEDS).*  *-No, I've probably become more comfortable with my body, in everyday life, than I was a few years ago, but I thrive best in safe surroundings, I don't come up with so many pranks, or many strange things. I'm with people I know well and who know the diagnosis, fortunately I have many friends who have a good understanding that I can't be on all sorts of weird (Man with MFS)*  *- I have a very strange relationship with my body, I think that my body is much older than myself, and that is the reason why I try to be a little active, my body withers, but should not wither so early, therefore I can push my body sometimes quite a lot because I feel it slipping away, every now and then, even though I don't feel sick, (Women with vEDS)*  *-I have this endless guessing game in my head, and the same happens when I do housework – is it really wise, but I need to keep the house clean. Before I got diagnosed, I never thought about it, just focused on what I could do, certainly not that I could die from it. To this day, I`ve had nothing with my heart, but I live with the ghost of heart issue all the time (Woman with LDS)*  *-I was probably happier before, then I didn`t have the host of heart issue, yeah, because I was sure I wouldn’t have heart problems in my life, no one in the family does, so now it`s something I always have to consider (Woman with LDS)*  *- I want to point out that, in other words, the psychological burden of actually knowing that I have such a serious diagnosis, it is burdensome and you have to be aware of it. If you are aware of it and process it, I think you can live a better life (Woman with MFS)* | -The interviews indicate that coping with fear related to the disease seems deeply personal and ongoing process, both for the patients and the relatives.  -It is no facet, what works for one person might not work for another and the strategies seems to change over time.  -The consequence of fear may lead to avoidance (ignoring symptoms or care plans) or hypervigilance (excessive worry about their health). Both strategies can be detrimental.  - For some, fear may also cause that they withdraw and isolate themselves. While others had found more effective coping strategies that helped them maintain and enhance social support networks, which seems crucial for emotional support and practical assistance.  -Some also described that connecting with others who are going through similar experiences can provide comfort, understanding, and practical tips for coping. |
| Being able to control and accept fatigue and pain. | *-I mean, there`s a difference between pain and pain, when it comes to danger, it`s important … I`m always in pain anyway. I wish I know how to handle it (Woman with MFS).*  *-I have to be aware that I have the body I do, with its limitations, so if I want and maybe am allowed to live a bit longer, then of course, I have to be careful (Young man with vEDS)*  *-The problem is I react so differently, others get well and being active helps them, but for me, a long walk is just exhausting, and afterwards, I`m just tired, I zone out and am nor really present, but it might be another diagnosis like fatigue or chronic fatigue syndrome, so it`s hard to know what`s what (Women with LDS)*  *- For me it is a problem, you get isolated sitting at home, and you get tired even though you have to push yourself a bit, you don`t always have to taste blood, feel you push in your temples, it`s not always necessary.(Women with MFS)*  *-I`ve held back for a long time because I`m afraid I`ll get extremely tired, because that`s been my experience, while other days it`s totally fine (Man with LDS).*  *-The disease takes a lot of time and space in my life, the thought around this with energy saving, what should I have space for and not have space for. A shopping trip can give me a lot of energy, a refill that I know I need, but then I know I can't take it anymore that day. And it is always a question of what I can manage and what I should prioritize. (Woman with MFS).*  *- I don't have so much pain that I go and think about them all day, but they are there all the time, and probably influence me to get tired more quickly, but that's how life is in general, it goes beyond life in general (Man with LDS)*  *- Yes, because it is this the time that is the most challenging, when the fatigue sets in, and it takes a lot of effort to cope with this pain also, (Male with LDS)*  *- That is precisely what makes it so difficult, that we never know what the form will be like the next day, and then it becomes so difficult to plan, when it varies so much (Man with MFS).*  *- During physical activity, I notice discomfort, migraines, pressure in the head, and a little stress on the picture. I should have taken of the head, or the picture. After the operation, has a dissection taken place, has something else happened, there is a leak, you get a lot of thoughts when you don't get the help and control you need, so it doesn't help to say that you should exercise, so it doesn't help (Young man with LDS).*  *-Receiving the diagnosis, you get confirmation that you're not just lazy and stuff like that (Young man with vEDS).*  *- It is this point of intersection between quality of life and how much one has to expose oneself to (Man with LDS)*  *- Yes, it is very illogical, the feeling of fatigue, I can wake up with it, yes it is completely impossible (Women with vEDS),*  *- It's like a backpack, it's with me everywhere the anxiety, it takes so much space (Man with vEDS)*  *- I depend on having my routines, rhythm for the day, I rest in the morning and then I rest after eating dinner. And when we had guests for the whole week at Easter, then I was terrified, because I didn't get the usual pattern (Female with MFS)* | -Being able to control and accept fatigue and chronic pain seems to enhance their ability to live a fuller, more engaged life.  -Some claimed that it`s about not letting these symptoms define their existence They tried to find ways to coexist with them, minimizing their impact, and focusing on what brings joy and fulfilment.  - Some described that they needed to learn how to balance activity and rest to prevent overexertion and manage fatigue.  -Understanding the causes and mechanics of pain and fatigue was described at helping both the patients and the relatives to make informed decisions about the care and lifestyle. |
| Maintaining active engagement with family and friends | *-Sometimes I feel like she's like a ticking bomb and I'm anxious all the time, it's important that we take care of each other (Male spouse to a women with LDS)*  *- What we know, we've read up on, googled ourselves until there aren't many others to talk to about it since it's so rare. So, my daughter and I talk a lot with each other then, and she is an incredible person who lives in the present, must distinguish between what we should take in and what we shouldn't, true, and I am very happy that she is who she is (Mother to an adolescent with vEDS)*  *- I do the heaviest physical lifting to relieve him, but now I've got arthritis so you'll sort yourself out soon (laughs a little and the others too), but he's terrible at paying attention to me then. We have to look after each other. (Female spource to male with LDS)*  *- My friends I had when I got sick, they just saw that I was lying there sick. They were very supportive when I got sick. I punctured my lung and was in the hospital for half a year (Man with MFS).*  *- It is important to find new points of reference in life, and find other things to put your life on, when you are unfortunate enough to lose someone close, so that you do not go down into the basement and stay there. And yes, it's terrible (Female spouse to male with vEDS),*  *- Sometime I think, what has she done to deserve it, and it is bloody unfair of fate, and we often ask ourselves the question if there is any meaning in it (Male relative)*  *- My family and friends means a lot for me, I could not do it without them. They make me laugh and see the brighter side of life, but sometimes it`s my turn to lift them up and make life bright and fun, so it`s not just worry and sadness getting us. Otherwise, there wouldn’t be much of a life (Woman with vEDS)*  *-There are so many other things that matter too. So she's someone who wants to live as normal as possible, but it's not the same life as before. For me, I find it very difficult (Male relative).*  *-He prefers not to talk so much about the illness and neither do I, We will live as normal. If it's meant to be, then it's meant to be and …you shouldn't lay down your life. It is the motto we both have that we should live as usual as possible, but take care (Female relative)*  *- So you learn to live a little differently and that is fine for me, and we endeavoured to always think like that. And that calms me down too (Female to male with LDS)*  *- The disease does not take up a lot of space, only the times the crisis arrive, then it takes up a lot of space - but usually when it's over, such crises and operations and such, then it takes little space again. We want to live as usual and plan trips and go to the mountains and work as usual (Male relative)*  *- More focus on trying to maintain normalcy in the family is important for me. I do not let the disease affect me more than necessary. Me and the kids we have a lot of gallows humour (Woman with LDS)*  *- We have a saying in the family - that life is not for amateurs", heh, heh, and I think we are pretty good at it (Woman with MFS)*  *- I feel so much support from my family, but at the same time, I feel they`ve taken on an extra burden because of me. They`re always scared something might happen to me, even though they say life feels much more intensive now. We never take life for granted anymore, so it`s like we`re always living on borrowed time, and that`s powerful (Woman with LDS)* | -Maintaining active engagement with family and friends can ensure a support network that seems vital for coping with the daily challenges of living with HTAD.  -Some claimed that having a family and good friends foster a sense of belonging, enhance quality of life.  - This belonging were described as emotional, practical, and emotional support and helped them to navigate life effectively and joyful.  -Some also emphasized that family and friends can offer practical helps, whether it`s assisting with daily tasks, navigating healthcare needs, or providing transport.  -Some described relation to family as a way of providing a sense of normalcy and provided antidote to stress and anxiety. |
| -Finding health-promoting physical activities | *-We try to get the physical activity done, then there will be some quality time afterwards on the sofa and Netflix (Female relative)*  *- Yes, the advice and restrictions, I am told that I would rather not do so much, but still you must do something, but no one has any answer, there is nothing concrete, no, no one has told me that and you can do that, that's fine (Young man with vEDS).*  *- I just have to try to keep my body in shape, but it will burn out if you use your body a little too much (Woman with vEDS),*  *- yes, that's an important point, because if I go on a ski trip one day, I don't get to do anything more that day than that ski trip, then you have to recharge yourself, there are limits to how much you can do (Man with LDS)*  *-The advices and recommendation for physical restriction is important, and I always ask for which activities I can do. I need to be active and do some physical activities (Man with MFS)*  *- Yes we received a lot of information, but it is not so easy to find the right activities, but I know that I shall not exercise hard, have low impact and moderate intensity activity, not higher level. It might be a bit boring some time, but I manage (Man with vEDS)*  *-It is easier when I have annual check-up of my heart and blood vessels, and everything is okay, then I feel more safe and motivated for exercise (Man with vEDS)*  *-* | -Finding health-promoting physical activities seems crucial for people with HTADs and their relatives.  -They realize that they needed to be cautious and seek medical advice regarding physical activities. It was important for them and their relatives to find safe and suitable ways to stay active. It seemed as a key component of managing their condition and enhancing their overall health and well-being.  - Some described it as important to consult healthcare providers to understand which activities are safe and recommend, as some may be restricted due to risk of aortic dissection or other complications.  -Regularly check-ups and monitoring seems also crucial to ensure that the physical activity is not adversely affecting their health.  -The interviews indicate that successfully engagement in physical activity can give them a sense of control and accomplishment.  -Most participants emphasized that needed tailored activities to their ability and health status.  -Most of the participants had received information and advices that activities with low-impact, moderate-intensity activities are most appropriate. |
| **Monitoring and meetings with Health services** |  |  |
| Feeling safe and cared for | *- You're alone as relative, and you have no one to talk to except then - yes, the surgeon who came and asked how I'm doing, you and stuff like that, then it would have been good if someone had grabbed me and taken me somewhere, and chatted with me! Not because I expressed that I needed it, but "come here and we'll talk together"-like telling them that now I need a really good hug, that is. And that you take care of me (Male spouse to female with vEDS)*  *- I was very surprised at how many times the surgeon who operated on my husband was talking to me – a feeling that he saw me – it surprised me and I think that was very positive and I really feel that I could trust him (Female relative)*  *-I feel helpless; our life has been completely turned upside down. And it's almost like I want to say that nobody knows anything about this, I feel that the healthcare system has failed us, so hopelessly – we need to feel safe and cared for. (Male relative).*  *-I feel so much responsibility, and I never know what happened. Nobody cares about me, so when my husband is sick, seriously sick, I have to wait for a telephone and hope he still is alive (Female relative)*  *- It is important to be able to balance, to be available and to be supportive, while at the same time taking care of myself (Female relative)*  *- The mental part is important, it`s what replenishes you. Just knowing you`re living with such a serious diagnosis, it`s burdensome, and then you have to be conscious about it. It you`re aware and work through it, and it helps with support from the health- and social system. Then we might try to live a better life” (Woman with LDS)*  *-Yeah, about having and not having trust to health care, it`s stressful and affects the inner self. We`re not supposed to have any stress, so it becomes even more stressful. It`s burdensome when I don’t feel like I`m getting the follow-up care I need from the hospital and keep meeting new doctors who don`t know my diagnosis. That`s a bit stressful, and I lose trust. (Man with vEDS)*  *-We need knowledge and it is important that we can trust the doctors and nurses, then we can talk to them and thereby 100% follow up their advices (Female relative)* | Feeling safe and cared for was not just comfort; it was described as a fundamental need that significantly impacts the psychological and physical journey of the patients and their relatives.  - The interview indicated that it was important about that the healthcare provided a compassionate and supportive environment that upholds dignity, fosters resilience, and enhance the quality of life particularly during the most challenging times.  -Knowing that they are in a safe and caring environment was described as reducing anxiety and depression for both the patient and their relatives.  -Some also claimed that when they feel supported, it is easier to make more informed and considered decisions regarding treatment options, care- and rehabilitations plan.  -The relatives described that they often take on significant caregiving roles. Feeling supported and knowing that their loved one is receiving good care seem to lessen the emotional and physical burden they face.  -Feeling safe and cared for may also build trust between patients, families, and healthcare providers. Trust seems crucial for effective communication and collaboration in care management. |
| -Being recognized, seen and accepted | *-Yeah, with advice and restrictions, I get told what I ideally shouldn`t do, but still, you should do something, but no answer, nothing concrete. I wish someone could tell me what I can do and not just what I shouldn’t do, that would be nice.(Man with LDS)*  *- It is not easy to be a relative, and it might not be easy for the health service to see our needs, but it is important that they recognize that we are an important part (Woman relative)*  *-I feel they can easily overlook our needs as relatives, and forget that we`re there, but they must not forget us, because our jobs as relatives are of great importance (Woman relative).*  *-For me, it`s incredibly important that they take the time to tell me what`s happening, and that I`m not just left to fend for myself. Some healthcare professionals are better than others, and I understand that they`re terribly busy and sometimes they can`t make the time. But I wish they could see us and accept our needs to information about our loved (Woman relative)*  *-I like it, when they are specific – when they can give me specific advices – and I ask – I like swimming and can I do it. For me, it`s amazing, it doesn`t stress my joints, and I can swim as fast as I want, that`s got to be better than doing nothing, so I hope I can get a clear answer (Man with vEDS).*  *-Sometimes, I wish I had something visible, not for pity, but for understanding and accept from others. That would be nice (Man with MFS)*  *-When my body dictates so much: it`s like I`ve lost all control. I have to obey my body more than it is listens to me. But I`m trying to find a balance in live that gives meaning, support and recognition from others is important (Woman with MFS)*  *-In our family, it is important that the diagnosis should not take all attention. I actually have a good relationship with my body, even though I can do less than before. I try to speak kindly to myself, and for me its fundamental that health professional recognize and accept my challenges when I talk to them (Women with MFS)*  *-I received a lot of information from several places at almost the same time. They’re supposed to follow up and take care of me, so I try to piece together all the information I received, trying to get a total understand of my disease, but I wish someone could have helped me in coordinating and understanding the fully picture of my disease (Man with LDS)* | When healthcare providers recognize, see and accept patients with HTADs and their relatives, it not only improves the emotional well-being of the patients and their families, some claimed that, it also was important for compliance and for the overall healthcare experiences.  -It seems that dealing with a life threatening disease as HTAD can be incredible stressful and for some it can lead to isolation and depression. Recognition and acceptance from healthcare providers may provide emotional support, reduce feeling of isolation, and improve overall mental well-being.  -Some claimed that when they felt understood and supported, they was more likely to communicate openly about their symptoms, concerns, and preferences.  -When patients and their families trust their healthcare providers, they are more likely to follow medical advice and treatment plans.  -The interviews indicate that trust is built through empathy, respect, and feeling understood, all of which may contribute to better adherence to treatments and medications. |
| -Factual and sober information and advices for management of the disease | *- I think the most important thing for me is to get precise and factual information, and if they don`t know, just tell me so (Man with LDS)*  *-It is easier to do as the doctor tells you if the information is understandable, concrete and with practical advices, so I can deal with it in the real world (Woman with vEDS)*  *-I think the doctors should prescribe a lot of discipline when you go from full training and exercise to drastically reducing it; it just doesn`t match up, and you lose motivation (Man with MFS).*  *-I often think healthcare professionals, general practitioners, and others who meet us in such difficult situation, they don`t understand our feeling and frustrations (Woman with LDS).*  *-For me it can be hard to take the bus, change trams, and walk many kilometres with a suitcase, but I look healthy and no one understand (Man with vEDS)*  *-Maybe we should get more information about the diagnosis, about how the blood vessels react and work when we lift, when we jump, or hold our breath. Getting help to understand what`s happening in the body, I think could help med be more realistic, without being terrified that I`ll die the next moment (Man with LDS)*  *-I think more factual information about all the vascular stuff in the body and how it functions should be illustrated, so we get more knowledge about it. So when I get a headache, I wonder if something dangerous is happening in my head, or is it just a regular headache, It can be hard to interpret such symptoms when we don`t have enough knowledge. And there should also be a program for anxiety, it`s exhausting and inhibiting (Man with vEDS)*  *- It was interesting that a few years ago, I started reading about the disease online, and it said the average age was 48 or 58 years for dying, but anyway, I was approaching that age, and it was a bit scary, so not all information is healthy (Women with vEDS)*  *- I was relieved to hear there were a lot of underreported cases, and that many live well and long with the diagnosis and many become 70 and 80 years old, It helped when I found some information about this (Women with vEDS).*  *-It was a shock, and I got anxious, but after all, it was better they gave me proper information rather than just making vague guesses. I think I would`ve been even more worried then. And it was nice that the information was a bit nuanced, that you can live a long and good life with the diagnosis, but there are some limitations (Man with MFS)*  *-What the professionals should advise is hard, especially when they say I can only lift 15 kilo, when they really don`t know. We need more individual knowledge about the diagnosis. Maybe we could keep a logbook of how we react to different thing, and then create our own approach and strategy (Woman with MFS).* | -Providing factual and sober information and advice seems vital for effective disease management, building trust and ensuring compliance with treatment. .  -Some claimed that factual information and understanding the nature of the disease, possible treatments, and potential outcomes may help them to actively participate their healthcare.  -Sober, honest information also seemed to help set realistic expectations about the disease`s progression, treatment efficacy, and possible side effects. Some claimed that this may help them prepare for the future, both practically and emotionally, and avoid false hope or misunderstanding.  -While the truth may sometimes be difficult to hear, the interviews indicated that uncertainty and misinformation is worst because it can lead to increased anxiety and fear.  -Clear, factual information seems alleviate unnecessary worries and allow patients and families to focus on coping and treatment. |
| -Increased Health literacy and support for coping. | *-What kind of advice professionals should give is difficult - but certainly don't give any advice exactly about kilos, that it's not what matters, but simply more knowledge about the diagnosis of the individual. Yes what can I say, status as awareness of, we are all aware that we have the diagnosis, if we take the signals that the body gives (Yong woman with LDS)*  *-It is easier to follow the recommendations and advices when they are understandable and easy to use in daily life (Woman with vEDS)*  *- I only remember one sentence "Am I running to fast for talking" - I remember that (Young man with MFS)*  *-When I can trust the doctors, and the advices seems reasonable, and they are listening to me, I feel we can cooperate, I think it is best for my health (Man with LDS)*  *- When I am prepared and have more knowledge about my diagnosis, I feel the doctors take me more serious, and it is easier to understand them, and they can easily understand me, then we understand each other, and I feel more safe and empowered (Man with vEDS)*  *- And then you start reading online, and that's not always a very good idea. Although eventually we only became even more depressed. So I don't know - I don't know what's the gentlest, best way to know, but that's how we experienced it at least. (Male relative)*  *-Sometimes I think if something increases my quality of life a lot, I`ll do it, even if I probably shouldn’t, and you never know the side effects, but I take the chance and it`s my choice (Man with MFS)*  *-It easier to take care of myself and my family if I have received factual of good information from the health service. It`s not easy to live without this type of information (Man with LDS).*  *- Maybe it's actually less dangerous than it is, maybe it's not as dangerous as you think - maybe the professionals really don`t know what's dangerous. Although you shouldn't hide under a chair that it's a serious diagnosis, but that most things we can do and maybe normalize it (Young man with MFS)*  *-It`s important to remember we`re much more than the diagnosis, more than pain and fatigue, even if these take a low of space. I try to push it aside sometimes, and live as much as possible (Man with MFS)*  *-There`s so much you can read on the internet, but I try to avoid it because there`s so much negativity, and you can pick what you want to read, but it is often the worst stories. (Women with LDS)*  *-When you search the internet, like if you`re going to have aortic surgery or eye surgery, the worst stories come up, and it terrifying. This doesn`t provide good knowledge, so we need sensible professionals to increase our realistic health competence. It is important to understand that not all information is useful. (Man with vEDS)*  *-I always say, we have to decide how much space the illness will take. Sometimes it`s all-consuming and gets all the attention, and sometimes we hardly notice it. It`s during those all-consuming sick periods that we need special support (Female relative)*  *-The thing about informing about the illness is double-edged and conflicting. I want to appear healthy but also need support. It can`t be easy to be the doctor who is informing (Woman with vEDS)*  *-I feel it is important that both me and my wife have enough knowledge to make serious decisions, but this assumes that we have access to realistic and useful information from healthcare professionals who know their field (Man with MFS)* | -The interview indicate that increased health literacy can able the patients to follow treatment plans, recognize symptoms, and understand when to seek medical help. Which seems important for effective management of the disease and quality of life.  -Higher health literacy may give the patients and their relatives a sense of control over their health journey, allowing them to actively participate in their care and advocate for themselves within the healthcare system.  -Health literacy can also improve patients` ability to communicate effectively with healthcare providers. Thereby, improving discussion about care, clearer understanding of medical advices, and more meaningful participation in healthcare decisions.  - Increased health literacy and support for coping seems essential for empowering patients and their families, improving disease management and communication, reducing stress, and enhancing the overall quality of life during a challenging time.  -The interview indicate that understanding the disease may reduce fear and empower the patients and relatives to make informed decisions about their care. However, it`s important to get information from reliable sources to avoid misinformation which can increase fear. |
